# Supplementary material for: Deciphering the combinatorial landscape of immunity
Source: eLife. 2020 Nov 23;9:e62148. doi: 10.7554/eLife.62148 (PMC7748411; doi:10.7554/eLife.62148)
Supplement: Transparent reporting form [file elife-62148-transrepform.docx]

***eLife’s* transparent reporting form**

We encourage authors to provide detailed information *within their submission* to facilitate the interpretation and replication of experiments. Authors can upload supporting documentation to indicate the use of appropriate reporting guidelines for health-related research (see [EQUATOR Network](about:blank)), life science research (see the BioSharing Information Resource), or the [ARRIVE guidelines](about:blank) for reporting work involving animal research. Where applicable, authors should refer to any relevant reporting standards documents in this form.

If you have any questions, please consult our Journal Policies and/or contact us: [editorial@elifesciences.org](about:blank).

**Sample-size estimation**

- You should state whether an appropriate sample size was computed when the study was being designed
- You should state the statistical method of sample size computation and any required assumptions
- If no explicit power analysis was used, you should describe how you decided what sample (replicate) size (number) to use

Please outline where this information can be found within the submission (e.g., sections or figure legends), or explain why this information doesn’t apply to your submission:

Sample size estimation analysis does not apply to our study, since majority of the analyses were performed on public data. The gene expression data produced specifically for this manuscript (IFNb, TNFa treatment of human monocyte derived dendritic cells) used the design of three replicates for each condition that is most commonly observed in high throughput expression data, striking a compromise between cost and replication.

**Replicates**

- You should report how often each experiment was performed
- You should include a definition of biological versus technical replication
- The data obtained should be provided and sufficient information should be provided to indicate the number of independent biological and/or technical replicates
- If you encountered any outliers, you should describe how these were handled
- Criteria for exclusion/inclusion of data should be clearly stated
- High-throughput sequence data should be uploaded before submission, with a private link for reviewers provided (these are available from both GEO and ArrayExpress)

Please outline where this information can be found within the submission (e.g., sections or figure legends), or explain why this information doesn’t apply to your submission:

1. High throughput gene expression data. GEO accession and reviewer token are provided with the submission. Experimental details, including replication and QC, are provided in the Materials and Methods section, under the subheadings *DC differentiation* and *Microarray data of human moDC treated with IFNβ and TNFα.* No outliers were observed.
2. Validation experiments for VCAM-1 and CCL-5 were also done in triplicate. Experimental details are provided in the Materials and Methods section, under the subheadings *DC differentiation* and *Experimental validation of IFNβ and TNFα synergistic effects.*

**Statistical reporting**

- Statistical analysis methods should be described and justified
- Raw data should be presented in figures whenever informative to do so (typically when N per group is less than 10)
- For each experiment, you should identify the statistical tests used, exact values of N, definitions of center, methods of multiple test correction, and dispersion and precision measures (e.g., mean, median, SD, SEM, confidence intervals; and, for the major substantive results, a measure of effect size (e.g., Pearson's r, Cohen's d)
- Report exact p-values wherever possible alongside the summary statistics and 95% confidence intervals. These should be reported for all key questions and not only when the p-value is less than 0.05.

Please outline where this information can be found within the submission (e.g., sections or figure legends), or explain why this information doesn’t apply to your submission:

Raw data is presented in figures. Statistical tests used, values of N, p-values, methods of multiple testing correction and dispersion measures are noted, where appropriate, in figure legends.

(For large datasets, or papers with a very large number of statistical tests, you may upload a single table file with tests, Ns, etc., with reference to sections in the manuscript.)

**Group allocation**

- Indicate how samples were allocated into experimental groups (in the case of clinical studies, please specify allocation to treatment method); if randomization was used, please also state if restricted randomization was applied
- Indicate if masking was used during group allocation, data collection and/or data analysis

Please outline where this information can be found within the submission (e.g., sections or figure legends), or explain why this information doesn’t apply to your submission:

Not applicable to this submission.

**Additional data files (“source data”)**

- We encourage you to upload relevant additional data files, such as numerical data that are represented as a graph in a figure, or as a summary table
- Where provided, these should be in the most useful format, and they can be uploaded as “Source data” files linked to a main figure or table
- Include model definition files including the full list of parameters used
- Include code used for data analysis (e.g., R, MatLab)
- Avoid stating that data files are “available upon request”

Please indicate the figures or tables for which source data files have been provided:

VCAM-1 and CCL-5 validation experiments data are included as additional data files. An interactive R Shiny application of iSAIL can be found at https://isail.shinyapps.io/test_app/. The site also contains

downloadable R code, models, parameters and documentation to run the software locally.
